# Rehabilitation and reintegration programming adjunct to female genital fistula surgery: A systematic scoping review

**DOI:** 10.1002/ijgo.13039

**Published:** 2020-01-13

**Authors:** Alison M. El Ayadi, Caitlyn E. Painter, Alexandre Delamou, Jill Barr‐Walker, Abner Korn, Susan Obore, Josaphat Byamugisha, Justus K. Barageine

**Affiliations:** ^1^ Department of Obstetrics, Gynecology and Reproductive Sciences University of California San Francisco San Francisco CA USA; ^2^ Department of Obstetrics and Gynecology Urogynecology Division Kaiser Permanente Oakland CA USA; ^3^ Department of Public Health Gamal Abdel Nasser University Conakry Guinea; ^4^ ZSFG Library University of California San Francisco San Francisco CA USA; ^5^ Department of Obstetrics and Gynecology Mulago National Referral and Teaching Hospital Kampala Uganda; ^6^ Department of Obstetrics and Gynecology Makerere University College of Health Sciences Kampala Uganda; ^7^ Department of Maternal and Child Health Uganda Christian University Mukono Uganda

**Keywords:** Female genital fistula, Fistula surgery, Rehabilitation, Reintegration, Scoping review, Supportive care

## Abstract

**Background:**

Female genital fistula is associated with significant physical, psychological, and economic consequences; however, a knowledge and practice gap exists around services adjunct to fistula surgery.

**Objectives:**

To examine rehabilitation and reintegration services provided adjunct to genital fistula surgery, map existing programming and outcomes, and identify areas for additional research.

**Search strategy:**

We searched the published and grey literature from January 2000 to June 2019. Two reviewers screened articles and extracted data using standardized methods.

**Selection criteria:**

Research and programmatic articles describing service provision in addition to female genital fistula surgery were included.

**Data collection and analysis:**

Of 3047 published articles and 2623 unpublished documents identified, 26 and 55, respectively, were analyzed.

**Main results:**

Programming identified included combinations of health education, physical therapy, social support, psychosocial counseling, and economic empowerment, largely in sub‐Saharan Africa. Improvements were noted in physical and psychosocial health.

**Conclusions:**

Existing literature supports holistic fistula care through adjunct reintegration programming. Improving the evidence base requires implementing robust study designs, increasing reporting detail, and standardizing outcomes across studies. Increased financing for holistic fistula care is critical for developing and supporting programming to ensure positive outcomes.

## Abstract 2

Holistic adjunct reintegration programming accompanying female genital fistula surgery is recommended; however, efforts to develop robust evidence on best reintegration practices are necessary.

## 1 INTRODUCTION

Female genital fistula is a debilitating traumatic injury affecting up to 2 million women, mostly in sub‐Saharan Africa.^1^ Annual global incidence may reach 100 000. Primary etiologies include pressure necrosis from prolonged and neglected obstructed labor combined with delay in accessing comprehensive emergency obstetric care, iatrogenic causes (i.e. during cesarean delivery or hysterectomy), and trauma. Prolonged obstructed labor is most prevalent; however, an increasing proportion of genital fistula is iatrogenic.^2^, ^3^, ^4^, ^5^

Women with fistulae experience uncontrollable leakage of urine and/or feces, resulting in genital sores and infection.^6^, ^7^ In addition to pain and general weakness,^8^ women may experience nerve damage, uterine cervix injuries, and pelvic bone trauma that present as secondary infertility and gait disorders.^9^ Most babies involved in fistula‐causing deliveries do not survive.^10^ Women with fistula are stigmatized, restricted in social and economic participation,^8^, ^10^ and report high psychiatric morbidity including depression, which may persist even after surgical repair.^11^, ^12^, ^13^, ^14^

Access to genital fistula surgery has improved in sub‐Saharan Africa and many women experience improvements in physical and mental health following fistula repair alone; however, numerous women face continued physical and psychological challenges to resuming prior roles or adjusting to new circumstances. They may require further medical care depending on injury severity and surgical outcomes, and medical support for subsequent pregnancies and births. Longitudinal studies from sub‐Saharan Africa have identified concerning adversity following fistula surgery, including fistula recurrence, persistent fistula‐related symptoms, subsequent fertility challenges, and adverse perinatal outcomes.^15^, ^16^, ^17^, ^18^, ^19^ In Guinea, 16% experienced fistula recurrence by 24 months.^17^ In Uganda, by 12 months following repair, one‐third had persistent urinary incontinence, 17% weakness, and 9% general pain.^20^ In Malawi, only one‐fifth of women with reproductive potential became pregnant in the year following surgery.^16^ Experience of persistent physical adversity correlates with substantially lower psychosocial health.^20^ Such factors limit women's ability to resume previous roles despite successful surgery, particularly in conjunction with economic hardship,^21^ resulting in additional reintegration needs.^22^, ^23^

A knowledge and practice gap exists around women's postsurgical reintegration programming. Preliminary evidence supports short‐term facility‐based psychological intervention.^24^, ^25^ Physical therapy has also been recommended,^26^ as has improvement of economic independence.^27^, ^28^ Research synthesis on the reintegration process, evaluation, and service provision is important for developing evidence‐based service prioritization to meet the health needs of women recovering from genital fistula. Thus, the objective of this scoping review was to examine the range of rehabilitation and reintegration services provided as adjunct to genital fistula surgery, map the existing programming and outcomes, and identify areas where additional research is necessary.

## 2 MATERIALS AND METHODS

Four research questions were specified to meet these objectives:

1. What rehabilitation and reintegration services are provided as adjunct to genital fistula surgery for women with obstetric, iatrogenic, or traumatic fistula?
2. What are the components of each rehabilitation and reintegration intervention, and how are they delivered?
3. What is the impact of each rehabilitation and reintegration intervention on women's physical, psychosocial, and economic status?
4. What are the study authors’ recommendations for rehabilitation and reintegration interventions and intervention delivery?

Our scoping review methodology followed Arskey and O'Malley^29^ and Levac et al.^30^ frameworks and Preferred Reporting Items for Systematic Reviews and Meta‐Analyses (PRISMA‐ScR) guidelines.^31^ The full protocol for this review is published in detail elsewhere and summarized herein.^32^

We searched the published and unpublished (“grey”) literature to broadly capture reintegration programming data. The search strategy was developed collaboratively with a medical librarian (JBW) with training and experience in systematic reviews using an iterative process including term harvesting, text and MeSH term extraction, and testing.^33^ The final search strategy was peer reviewed by a second librarian following Peer Review of Electronic Search Strategies guidelines.^34^ We searched reference lists of included articles and contacted authors for additional detail.

The published literature search strategy combined two main concepts: obstetric fistula and social reintegration, using Boolean logic. Our search was implemented on September 27, 2018 and updated on July 8, 2019 in PubMed (1966–), Embase (1947–), Popline (1970–), PsycINFO (ProQuest, 1887–), Web of Science (1900–), Sociological Abstracts and Social Services Abstracts (ProQuest, 1963– and 1980–, searched together), and African Journals Online (2004–) databases. The search strategy is available as supporting information Table S1. No language limits were used; however, we included only articles published from 2000 onward.

The grey literature search employed targeted Google (Google LLC, Mountain View, CA, USA) searches directly from organizational websites identified by UNFPA's Campaign to End Fistula (supporting information Data S1), and personal queries with other known clinical or social service organizations, implemented on June 16, 2019. The final search strategy was fistula and reintegration or rehabilitation or program or service, and was operationalized through Google Custom Search JSON API that imposed a 100‐result limit per website. We also searched available abstract books from the various conferences of three organizations: The International Society of Obstetric Fistula Surgeons (ISOFS); The Global Maternal Newborn Health Conference; and Women Deliver.

Titles and abstracts of published articles were independently screened by two reviewers (AE and CP) followed by full‐text screening and data extraction. A third reviewer (AD) was available to resolve discrepancies; however, none arose. In accordance with established scoping review frameworks,^29^, ^30^ critical appraisal of study quality was not performed. Inclusion and exclusion criteria are detailed in Table 1. Data from eligible studies were systematically extracted (Table 2). Where articles were eligible but lacked detail, data were summarized narratively. Unpublished studies and reports were screened by one of four reviewers (AE, CP, RB, LL); results are reported following a simplified PRISMA sequence in narrative format^35^

**Table 1:** Population, concept, and context for identification of eligible studies

Criteria	Description
Population	Females undergoing surgery for genital fistula Fistula etiologies: obstetric, iatrogenic (non‐cancer), traumatic All ages
Concept	Any research or service provision in addition to surgery; no limitations on intervention type Clinical or patient‐reported outcomes beyond surgical success Studies or reports including original research or program data
Context	All contexts; articles written in English or French

**Table 2:** Study details, published literature

Study (author/year)	Article type	Study objective	Country	Study design	Study dates	Participants	Intervention description (summary, location, duration, mechanism, structure, comparison)	Study outcomes and measures	Results	Recommendations (authors’ recommendations based on findings/experiences)
Castille et al.^38^, ^39^ (2014 and 2015)	Empirical research	To measure the impact of a physiotherapy and health education intervention on surgical repair outcomes	Benin	Quasi‐experimental (nonrandomized control and pre/post)	Control: Nov. 2009–Jan. 2011 Intervention: Mar. 2011–Mar. 2012 1‐yr follow‐up: Mar 2012–Mar 2013	*14 d*: 211 women with VVF (112 intervention, 99 control) *1 yr*: 84 women in intervention group, not lost to follow‐up	*Summary of components*: Health education and physiotherapy *Location*: Fistula repair camp *Implementer*: physiotherapists, nurses (follow‐up) *Duration*: Preoperative through 14 d postsurgery *Mechanism*: Maintain low abdominal pressure through activity modification *Structure*:* Preoperative*: 2–3 physiotherapist‐led didactic sessions to learn techniques to reduce abdominal pressure during daily activities including perineal contractions, hypopressive exercises, and behavioral instruction. *Postoperative*: Further physiotherapy sessions. Activity prohibitions for 3 mo: sitting up from back lie, twisting body, bending at abdomen, lifting with bend in back, sexual intercourse. Socially isolated women offered vocational training and/or microcredit (no additional detail). Follow‐up at 3, 6, and 12 mo (unclear if any additional instruction offered). *Comparison*: standard of care (no follow‐up)	*14 d*: Repair success (fistula repaired with or without incontinence vs failed repair or urinary diversion) *1 yr*: Quality of life (QOL; Ditrovie scale, range 10–50)	*Repair success (14 d)*: Repair success was significantly higher in intervention group (68.8% vs 57.6%; OR 2.72, 95% CI, 1.30–5.93, *P*=0.005). Number of prior surgeries also significant predictor of surgical repair success. Among women with closed fistula, urinary stress incontinence was lower in intervention group (52.6% vs 22.1%, *P*<0.001) *Repair success (1 yr)*: Among women with failed repair, 3/29 were healed after 1 yr. Among women with successful repair and USI, 6/17 had no USI at 1 yr. Among women with successful repair and no USI, 2/60 had fistula recurrence and 4/60 developed USI *Quality of life (1 yr)*: Mean QOL at surgery through 1 yr decreased from 36.3 to 13.0 among women with successful surgery and no USI, from 34.3 to 17.0 among women with successful surgery and USI, and from 38.7 to 29.4 among women with failed repair (including 10.3% who had achieved repair by 1 yr)	Health education and simple physiotherapy including pelvic floor training and abdominal wall management can reduce surgical failures and improve surgical repair outcomes for women with VVF. Positive impact was maintained for 1 yr following surgery, with substantial improvements in QOL. Overall care of women with be improved by adequate nursing and support by a trained physiotherapist
Johnson et al.^25^ (2010)	Empirical research	To assess the impact of a health education and psychosocial counseling program on fistula knowledge, self‐esteem, and behavioral intentions following surgery	Eritrea	Quasi‐experimental (pre/post)	Feb.–Mar. 2006	43 women seeking fistula repair	*Summary of components*: Health education and psychosocial counseling *Location*: Health facility *Implementer*: Nurses and public health workers *Duration*: 4 d (1 d preoperative through 3 d postoperative) *Mechanism*: Educating clients about fistula and prevention, building their self‐esteem and helping to prepare them for social reintegration will increase awareness about women's bodies, prevent postsurgical complications, and contribute to fistula prevention *Structure*: 2 individual counseling sessions (1 preop, 1 postop). *Preoperative*: counseling on fistula causes and prevention, stories of other women with fistula, client's specific fistula type and surgery, treatment scope and anticipated outcomes, postoperative recovery expectations, operative and postoperative complications, encouragement to consider life and health goals following repair. *Postoperative*: counseling on patient postop condition management, options if repair is unsuccessful, reproductive health and rights, family planning, personal hygiene, nutrition, medical follow‐up, community and familial reintegration, encouragement to share information with women in home community on fistula and safe motherhood, ideas about how to share information with others on fistula and fistula prevention *Comparison*: Not applicable	Fistula knowledge, self‐esteem (range 0–30), and behavioral intentions for health maintenance and social reintegration Focus groups (2, with 19 total clients) to explore patient experiences with surgical care and counseling	*Fistula knowledge*: significant increases in knowledge of what fistula is (45.7% to 79.1%), what causes fistula (fistula caused by lack of SBA 34.8%to 88.4%), whether fistula is preventable (strongly agreed preventable 37.0% to 90.7%, agreement with 4 + ways to prevent fistula 52.2% to 90.7%), and knowledge about surgical risks (19.6% to 51.2) *Self‐esteem*: significant increase in mean self‐esteem score (13.6 to 27.9) *Behavioral intentions after surgery*: significant increases in intent to use family planning (0.0%to 33.3%), intent to improve hygiene (13.0% to 44.2%), intent to improve nutrition (17.4% to 58.1%), plan to talk to family members about fistula (26.1% to 90.7%), and plan to talk to community members about fistula (34.8% to 76.7%) *Qualitative findings*: women felt prepared for what to expect for surgery after preoperative counseling, and shared that they felt better about themselves and their futures following treatment and counseling. Several women felt their family members should be counseled on fistula and fistula prevention	A formal counseling program can have significant positive short‐term impact on fistula patients through increasing knowledge about fistula and improving self‐esteem. Counseling programs should involve family members and provide information on and access to family planning methods
Keyser et al.,^40^ Keyser et al.^41^ (2014)	Empirical research	To describe components of a physical therapy for women with fistula and report on outcomes	Democratic Republic of Congo	Quasi‐experimental (pre/post)	May 2010–April 2012	205 total, 142 with discharge evaluation	*Summary of components*: Health education and physiotherapy *Location*: Health facility *Implementer*: Physical therapists and nurses *Duration*: 7–14 d of physical therapy, average of 9.45 sessions *Mechanism*: Educating clients about fistula and prevention, building their self‐esteem and helping to prepare them for social reintegration will increase awareness about women's bodies, prevent postsurgical complications, and contribute to fistula prevention *Structure*: Physical therapy sessions started at day 14 postoperatively *Comparison*: Postsurgery compared with hospital discharge	Pelvic floor muscle strength (range 0–5), contraction endurance (contraction time), contraction repetitions, fast contractions, level of continence (self‐report, Addis Ababa Fistula Hospital Incontinence Scale; range 1–5).	*Pelvic floor muscle strength*: mean 2.45 postop to 2.54 at discharge, with 17.6% improving in grade *Contraction endurance*: mean 1.83 postop to 2.25 at discharge, with endurance improving among 45.5% and worsening among 5.6% *Contraction repetitions*: mean 5.40 postop to 8.75 at discharge *Fast contractions*: 24.6% had change in number of fast contractions, with 21.8% improving and 2.8% worsening *Incontinence*: 63.4% had no incontinence at discharge. 21 women had change in reported continence level postop to hospital discharge; of these, 71.4% improved and 28.6% worsened (all those who worsened had repair failure)	Preliminary results suggest that integrated physical therapy for women undergoing fistula repair is feasible and may be an important adjunct treatment given observed improvements in pelvic floor functional capacity with limited exposure to physical therapy. Challenges to implementation included need for more support and continuing education in pelvic floor physical therapy, difficulty in following patients long term, high staff turnover, and limited funding for program continuation
Ojengbede et al.^24^ (2014)	Empirical research	To determine the impact of group psychological therapy on the mental health of obstetric fistula patients	South Sudan	Quasi‐experimental (pre/post)	Oct.–Nov. 2008	60 women	*Summary of components*: Health education and psychosocial counseling *Location*: Fistula camp *Implementer*: Trained nurse psychologist or clinical counselor *Duration*: 1 session *Mechanism*: Group interpersonal therapy among patients with similar mental health conditions helps patients to understand their health problems through sharing of information, experiences, and coping strategies *Structure*: Group sessions had 9–12 participants and lasted 45–60 min. Discussion topics included the cause of their health challenge, initial reaction of their family members and community to incontinence, how they have been able to live and interact with their community, and the emotional impact. Counselors guided participants to think of ways to solve problems. Sessions concluded with fistula education and dispelling myths *Comparison*: Presurgery compared with hospital discharge (14–21 d postoperative)	Depression, self‐esteem, and suicidal ideation	*Depression*: Depression significantly decreased from 71.7% to 43.4% *Self‐esteem*: Very low self‐esteem significantly decreased from 65.0% to 18.3% *Suicidal ideation*: Severe suicidal ideation significantly decreased form 15% to 0%, and no suicidal ideation significantly increased from 43.3% to 73.3%	Given the systematic reductions in all mental health measures after group psychological therapy, psychological counseling is recommended as an adjunct to surgical repair. Group psychotherapy offers the opportunity for individuals to share experience and coping strategies and is cost‐efficient in countries with limited human resources
Pollaczek et al.^45^ (2017)		To describe holistic fistula outreach, treatment and reintegration program content	Kenya	Program report and postparticipation mixed‐methods evaluation	Ongoing program; evaluation March 2012	40 support group members, with mean time since fistula surgery 2 y	*Summary of components*: Psychosocial counseling, social support, and economic empowerment *Location*: Hospital and community *Implementer*: Psychologists/social workers (counseling) and community representatives *Duration*: Not specified. *Mechanism*: Economic empowerment will help women resume activities in community life and have greater agency and access to resources through: (1) challenging ideologies that justify social inequality; (2) changing prevailing patterns of access over resources; and (3) transforming institutions and structures that reinforce and sustain existing power structures *Structure*: Three‐pronged approach: (1) outreach/identification of women with fistula and referral to care; (2) surgical repair and psychosocial counseling; and (3) reintegration assistance. At repair hospitalization, women undergo 2–3 individual or group psychosocial counseling to help women work through anxieties. Women are then escorted home by program staff and linked to peer support groups. Groups provide psychosocial support, teaches income generating skills and women participate in group‐led income generation activities. Group participation increases access to bank accounts and microloans. Women and their families are enrolled in the Kenya National Hospital Insurance Fund *Comparison*: None	Emotional well‐being, fistula knowledge, and economic status	*Well‐being*: 90% reported support groups helped them a lot to make new friends, communicate with family members, and feel happy in lives *Fistula cause*: 85% indicated support groups helped them a lot to understand causes of fistula. Women in FGDs were able to articulate fistula causes and how to prevent recurrence after repair *Economic status*: 9% reported that they had been helped a lot with their basic needs, 64% said helped somewhat, and 23% said helped not very much. FGD participants reported success in developing income generating projects, getting larger loans out from the bank and respect in the community	Fistula survivors trained to serve as community‐based representatives can effectively improve community awareness, increase identification of women with fistula and refer to surgical and psychological treatment, and provide meaningful reintegration assistance. A holistic integrated model of outreach, treatment, and reintegration can have a large impact on women's lives, physically and emotionally. In time, it may also help women financially. Such a group model may be most effective in areas where fistula is concentrated.
Trombley and McKay^36^ (2010) Program also reported on in Diallo^37^ (2009)	Program report	To describe fistula‐related programming in Guinea funded by USAID	Guinea	Program report	Report on programming 2008–2010	Women with genital fistula attending Kissidougou repair hospital; no details provided	*Summary of components*: Health education and social support *Location*: Community *Implementer*: Waiting home coordinator, host families *Duration*: Not specified *Mechanism*: Social immersion in nonjudgmental environments supports women's recovery and reintegration. Health education and communication skills will help women become agents of change in their communities *Structure*: Women stay in a waiting home for 2 wk prior to their surgery for testing and intake processes, and return to this waiting home after hospital discharge (14 d after surgery) until they have recovered. The coordinator supports patient morale and helps to create a sense of community at the waiting home. At the waiting home, women receive health education, orientation to publicly speaking, and interpersonal communication training. For women who have a longer recovery period or who would have difficulty returning for a 3‐mo postoperative check‐up, a host family initiative has been developed. Staying with a host family helps facilitate the transition between the waiting home and return to home villages, as women are able to participate in normal family activities to the extent to which they are comfortable *Comparison*: None	Participant engagement in community educational sessions	Participant community engagement: 53% of women who stayed at the waiting home had conducted at least 1 awareness session in communities Diallo: anecdotally, women experienced increased confidence, self‐esteem, and emotional health	Waiting home model provides physical and social support to women. Greater attention is needed to address the psychosocial needs of patients and improve knowledge of host families. Other skills‐building or income‐generation activities would further facilitate patients’ reintegration; however, no funding is currently available for this
Watt et al. 42, 43 (2015), Watt et al.^44^ (2017)	Empirical research	To develop a mental health intervention for obstetric fistula patients, and pilot test for feasibility and acceptability	Tanzania	Intervention development and pilot study	Feasibility/acceptability pilot: 2014 (not further specified) Pilot RCT: Mar 2014–June 2016	Feasibility/acceptability pilot: 6 fistula patients Pilot RCT: 60 women, 30 intervention, 30 control	*Summary of components*: Psychosocial counseling and health education *Location*: Hospital *Implementer*: Trained community health nurse *Duration*: 6 individual psychotherapy sessions over a 2‐wk period (2 presurgery, 4 postsurgery) *Mechanism*: An intervention based on CBT and coping skills will lead to effective coping and improved mental health, further resulting in social well‐being and general functioning, and improving ability to reintegrate *Structure*:* Session 1*: Normalize patient's experience, acknowledge fistula impact, explore fistula influence on self‐perception, generate therapy goals, and learn relaxation exercise (to practice daily). *Session 2*: Practice relaxation exercise, review any other assignments, cognitively reframe fistula experience through education, counsel on fistula surgery, discuss treatment hopes/anxieties/questions. Patients assigned relaxation practice and asking healthcare provider questions. *Session 3*: Introduce to cognitive model, begin teaching how to reframe negative/unhelpful thoughts, teach ‘serenity prayer’ and relate to individual coping. Patient assigned relaxation practice and practice reframing negative thought. *Session 4*: Help patient recognize and respond to stressors using appropriate and effective coping skills, create behavioral plan for coping with negative stressors. Patient assigned coping strategy practice. *Session 5*: Examine the effect of social relationships on patient's life, generate specific strategies to strengthen social relationships, role play potential discussions about fistula, facilitate call between patient and support person to facilitate return home. Patient assigned consideration of thoughts and feelings about going home. *Session 6*: Prepare the patient to return home, discuss patient thoughts/emotions, problem solve how to handle circumstances and cope with potential stressors, develop a detailed action plan, facilitate second phone call with support person to share reintegration plans and solidify support, share summary of postsurgery medical recommendations (e.g. 3 mo of sexual abstinence, no heavy work), review goals, achievements, and plan *Comparison*: None (feasibility/acceptability pilot), standard of care (pilot RCT)	Feasibility/acceptability pilot: Patient satisfaction with intervention, counselor, number of sessions, and time spent per session Pilot RCT: Patient satisfaction with intervention, counselor, number of sessions, and time spent per session, depression, anxiety, PTSD, self‐esteem	Feasibility/acceptability pilot: *Satisfaction with intervention*: 5/6 very satisfied; 1/6 satisfied *Satisfaction with counselor*: 6/6 very satisfied *Satisfaction with number of sessions*: 4/6 right amount, 2/6 too much *Satisfaction with time spent per session*: 4/6 right amount, 2/6 too much Pilot RCT: *Depression*: Mean symptoms dropped from 25.9 to 12.4 in control and 22.1 to 9.9 in intervention from baseline to post‐treatment (n/s), and to 6.6 in control and 6.4 in intervention at 3 mo (n/s) *Anxiety*: Mean symptoms dropped from 10.8 to 2.6 in control and from 10.6 to 2.4 in intervention from baseline to post‐treatment (n/s), and to 1.0 in control and 2.1 in intervention at 3 mo (n/s) *PTSD*: Mean symptoms dropped from 38.8 to 31.1 in control and from 37.1 to 28.6 in intervention (n/s) from baseline to post‐treatment, and to 23.8 in control and 26.2 in intervention at 3 mo (n/s) *Self‐esteem*: Mean self‐esteem increased from 12.4 to 21.9 in control and 13.9 to 23.6 in control from baseline to post‐treatment, and to 24.0 in control and 25.0 in intervention at 3 mo (n/s) *Satisfaction with intervention*: 30/30 would take part again *Satisfied with intervention*: 96.7% *Satisfied with intervention facilitator*: 100% *Satisfied with number of sessions*: 80% (20% said too many) *Satisfied with time spent at sessions*: 76.7% (23.3 said too long)	Formative work to tailor and adapt psych theory was critical. Integration into clinical flow, and feasibility for using a nonspecialist mental health person. If this intervention should prove efficacious in a future randomized control trial, then scale up within fistula repair programs should be considered Women presenting for obstetric fistula surgery have high levels of distress. A nurse‐led mental health intervention is feasible and acceptable, with high rates of fidelity and was rated positively by participants. Successful surgical repair has a big outcome on postrepair mental health outcomes. The study potentially suggests that women with unsuccessful surgeries may particularly benefit from an intervention focused on cognitive reappraisal and coping behaviors, including the potential for home‐based follow‐up. Addressing the accumulated psychological and social effects provides holistic care

Abbreviations: VVF, vesicaovaginal fistula; USI, urinary stress incontinence; SBA, skilled birth attendant; FGD, focus group discussion; RCT, randomized controlled trial; CBT, cognitive behavioural therapy; PTSD, post‐traumatic stress disorder.

## 3 RESULTS

### 3.1 Published articles and program reports

**Figure 1:**
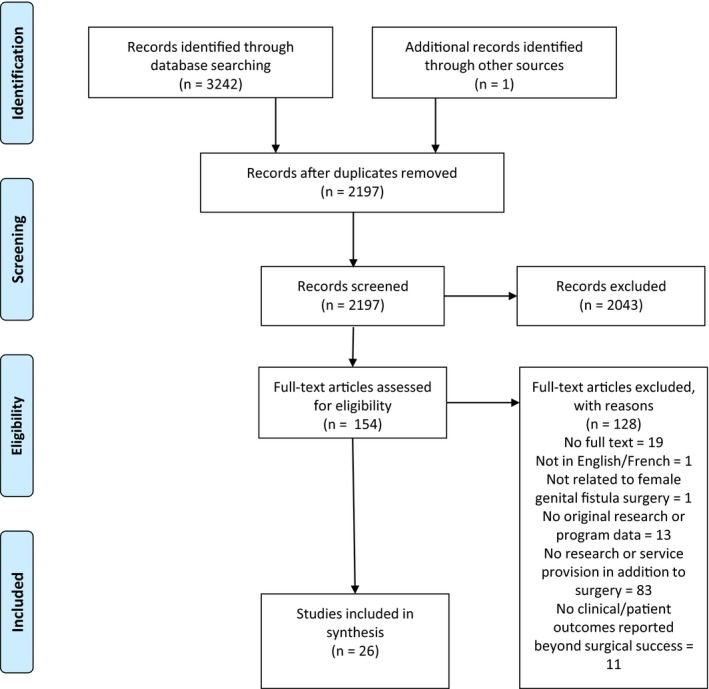
PRISMA flow diagram for selection of eligible studies and program reports. From: Moher D, Liberati A, Tetzlaff J, Altman DG. Preferred reporting items for systematic reviews and meta‐analyses: the PRISMA statement. Bmj 2009; 339: b2535.

Our database search identified 3242 articles. Excluding duplicates, 2197 articles were screened; 25 were found eligible and one related article was identified during web searching of article PDFs. The modified PRISMA flow diagram for selection of available studies is given as Figure 1. Twenty‐six articles were analyzed. Among these, 11 articles representing seven different studies or programs were considered “primary,” including a meaningful level of detail on program components and outcomes, and are presented in the data extraction table (Table 2). Fifteen articles representing 10 different studies or programs were considered “supplementary,” meeting the eligibility criteria but lacking detail, and are summarized within a separate narrative section. Results from the same study or program are presented together in table and narrative. One program had one primary^36^ and one supplementary^37^ article; both are described within the primary results.

### 3.2 Primary articles

Primary studies or programs identified were from Benin, Democratic Republic of the Congo (DRC), Eritrea, Guinea, Kenya, South Sudan, and Tanzania. Various intervention component combinations were employed, including health education (n=4), physiotherapy (n=1), psychosocial counseling (n=3), social support (n=2), and economic empowerment (n=1).

#### 3.2.1 Rehabilitation and reintegration intervention components and delivery

Two studies tested a combined health education and physiotherapy program.^38^, ^39^, ^40^, ^41^ In Benin, Castille et al.^38^, ^39^ employed physiotherapist‐led didactic sessions to assist women in activity modification including perineal contractions, abdominal hypopressive exercise, and behavioral instruction. In DRC, Keyser et al.^40^, ^41^ sought to increase pelvic floor strength and prevent postsurgical complications through physical therapy starting 14 days after surgery.

Three studies investigated combined health education and psychosocial counseling programs. In Eritrea, Johnson et al.^25^ tested individual counseling sessions addressing fistula, general health, and nutrition knowledge; postoperative condition‐specific management and recovery expectations; and explored women's postrepair life and health goals. Compared to preassessment, fistula knowledge, self‐esteem, and behavioral intentions after surgery significantly increased. In South Sudan, Ojengbede et al.^24^ determined the impact of an interpersonal therapy session before surgery. From presurgery to hospital discharge, depression, self‐esteem, and suicidal ideation decreased significantly. In Tanzania, Watt et al.^42^, ^43^, ^44^ tested a 2‐week individual cognitive behavioral therapy intervention on mental health. Improvements in depression, anxiety, post‐traumatic stress disorder (PTSD), and self‐esteem from baseline to follow‐up were not significantly different by intervention group, but feasibility and intervention satisfaction were high.

One report, by Pollaczek et al.^45^ from Kenya, described an intervention comprising psychosocial counseling, social support, and economic empowerment. Individual counseling was followed by community‐based peer support group linkage for social support and economic empowerment through income‐generating activities. Most participants reported improvements in emotional well‐being and fistula‐related knowledge, and high intervention satisfaction; however, economic gains were modest.

One report described an intervention combining health education and social support.^36^, ^37^ In Guinea, women lived in a supportive group home or host family while recovering from surgery, and received health education sessions and public speaking and interpersonal communication training. The program sought to facilitate women's transition to family life while ensuring postoperative care access and prepared them as community educators. No outcome data were provided; however, anecdotally, participants experienced increased confidence, self‐esteem, and emotional health.^37^

#### 3.2.2 Outcomes of reintegration programming by program components

##### 3.2.2.1 Health education and physiotherapy

Outcomes from two interventions combining health education and physiotherapy were not directly comparable.^38^, ^39^, ^40^, ^41^ At hospital discharge, Castille et al. identified higher repair success among intervention participants (69.9% vs 57.6%) and persistent incontinence was significantly lower in the intervention group among women with closed fistula. Keyser et al. also reported a decrease in stress incontinence. Castille et al. reported improved quality of life by 12 months among women with closed fistula. Keyser et al. reported short‐term increases in pelvic floor muscle strength, contraction endurance, contraction repetitions, and fast contractions.

##### 3.2.2.2 Health education and psychosocial counseling

Outcomes from combined health education and psychosocial counseling interventions included self‐esteem (n=3) and depression (n=2). All three studies identified significant improvements in self‐esteem; in the only controlled study, no differential self‐esteem increase was found between intervention and control groups. Significant decreases in depression were identified by Johnson et al.^25^ and Watt et al.^42^, ^43^, ^44^; again, intervention differences were not significant. Other outcomes included increased fistula knowledge and healthy behavioral intentions,^25^ and significant reductions in severe suicidal ideation,^24^ anxiety, and PTSD symptoms.^45^ No difference between intervention and control groups was found for anxiety and PTSD symptom reduction.^44^

##### 3.2.2.3 Health education and social support

One intervention combined health education with social support^36^, ^37^; anecdotally, women experienced increased confidence, self‐esteem, and emotional health.

##### 3.2.2.4. Psychosocial counseling, social support, and economic empowerment

Outcomes of the combined psychosocial counseling, social support, and economic empowerment intervention suggested that women's emotional well‐being and fistula knowledge improved significantly with intervention participation.^45^ Economic status had modest gains, with most women reporting that their economic status had been helped somewhat by participating in the program (64%).^45^

##### 3.2.2.4 Outcomes by fistula etiology

Outcomes of reintegration programming were not reported by etiology of genital fistula.

#### 3.2.3 Authors’ recommendations

The authors’ recommendations from primary articles supported holistic programming at genital fistula surgery and extending to community, despite most study outcomes measured at short term. Most articles recommended increasing women's access to incorporated intervention components given formal or anecdotal findings. Psychosocial counseling for women^36^ and family members,^25^ access to family planning methods,^25^ and income‐generating activities^36^ were recommended.

Recommendations for intervention design and implementation were discussed. Watt et al.^44^ reported factors critical to the feasibility and acceptability of their intervention included a formative research phase to contextually adapt and revise intervention design, ensuring that the intervention integrated easily into clinical flow. Additionally, task‐shifting to nurse‐level facilitators improved pilot intervention feasibility, and was anticipated to improve adoption.^44^ Challenges to program implementation largely focused on sustainability factors such as continuing support and education in intervention modality (i.e. physical therapy),^41^ challenges to long‐term follow‐up,^41^ high staff turnover,^41^ and funding.^36^, ^41^

### 3.3 Supplementary articles

Fifteen additional articles representing 10 studies or programs were eligible but limited in information; most were conference abstracts. Bangser and Haile‐Mariam^46^ reported on the effectiveness of the USAID Ethiopia program, which included transport home, follow‐up, and cash or in‐kind support, and was valued by stakeholders. Donnelly et al.^47^ reported qualitative experiences of such programming; women received food, clothing, stipend (some), contraceptive counseling, and education on postrepair pregnancy care needs. Benski et al.^48^ reviewed a multidisciplinary strategy for fistula management in Benin on physical and social outcomes; however, the intervention was not described. El Ayadi et al.^49^ explored adjunct service receipt and reintegration score in Uganda; no intervention details were captured. Hagos and Abebe^50^ reported on an Ethiopian model including provision of sanitary materials, training in life skills and income‐generating activities, linkages with women's associations, and education to support fistula identification and referral. Jarvis et al.^27^ described skills training provided to northern Ghanaian women; anecdotally, trainings were beneficial, but some felt the skills taught were inappropriate for the market, personal physical circumstances, or otherwise insufficient to change economic status. Mohammad^51^ described a residential Nigerian program designed to improve health and socioeconomic status; however, no intervention description or outcomes were reported. In Tanzania, Mselle et al.^52^ described patient participation in a society for disabled individuals that provided health insurance, paid medical bills, and supported income‐generating activities. Parameshwar et al.^53^, ^54^ evaluated postoperative recovery in a Ugandan fistula camp using a group‐based model of postoperative care that integrated physical and psychosocial healing. The intervention was not described, but findings suggested support and hopefulness were increased among recipients. Finally, Shittu et al.^55^ described all Nigerian fistula hospitals as capable of rehabilitation/reintegration services including elementary education, skills acquisition, and counseling.

### 3.4 Unpublished articles and program reports

#### 3.4.1 Internet

Our grey literature search resulted in 2623 eligible links across 123 websites; 1805 functioning and downloadable in PDF or html format were screened, and 55 were analyzed. The level of detail was variable, with most broadly summarizing activities, and some programming reported by multiple partners. Where organizations provided services across multiple locations, it was difficult to discern site‐specific programming. Reintegration programming identified primarily included health education, psychosocial counseling, and skills development or business education for economic empowerment. Some included patient advocacy. Fewer discussed physical rehabilitation or contraceptive counseling. Most programming directly targeted women; although some extended counseling to families/partners or built community awareness and resources for support and stigma reduction. Findings lacked outcome data, with few documents presenting participant comments. Finally, programming dates were difficult to establish.

Multicomponent reintegration programming where adequate detail was provided is summarized in Table 3. Materials from UNFPA and EngenderHealth evidenced broad partnerships. UNFPA documents emphasized reintegration programming, specified as psychosocial and socioeconomic support, as a pillar of the Campaign, and facility‐based reintegration programs are tracked. Findings highlighted programming across multiple countries (Burkina Faso, Central African Republic, Chad, Ghana, Guinea, Kenya, Liberia, Madagascar, Niger, Nigeria, Republic of Congo, Sierra Leone, Sudan, Tanzania, and Zambia; also highlighted were early steps to developing programming in Eritrea and Ghana) including combinations of health education, psychosocial counseling, and income‐generating activity training, literacy, and business skills. However, programming is generally lacking, with few women benefitting where programming is available, and best practices in social reintegration are needed. EngenderHealth findings emphasized vocational training, physical rehabilitation, counseling and emotional support, and stigma reduction. Country‐focused needs assessments and other documents highlighted variability in available programming ranging from none (DRC), to some vocational training (Mali, Nigeria), some startup funding (Chad), some counseling (Niger), and some multicomponent programming (Guinea and Sierra Leone). Recommendations included development of holistic programming including social support, counseling, health education, and a focus on patient‐centered care models.

**Table 3:** Summary of multicomponent programming identified in grey literature review.^a^

Organization	Geography	Summary of programming
Beyond Fistula	Kenya	Counseling, vocational training, business training, and educational scholarships
Comprehensive Community‐Based Rehabilitation in Tanzania	Tanzania	Mabinti Center offers psychological counseling, family planning, and HIV/AIDS prevention in addition to a vocational training program in handicrafts that helps women to start their own businesses and become financially independent
Freedom from Fistula	Madagascar, Malawi, Kenya	Classes in literacy/numeracy, handicrafts, micro‐finance support and business; contraceptive counseling; seeks to develop patient advocates
Hamlin Hospital	Ethiopia	Comprehensive learning, health, and reintegration services including nutrition, physiotherapy, psychological counseling, and training in income‐generating skills
TERREWODE	Uganda	Postsurgical care, nutritional support, psychosocial counseling, life‐skills training, health education, and income‐generating skills development. TERREWODE develops patients as advocates, and builds individual and community resources for support and stigma reduction
WADADIA	Kenya	Health education, psychosocial support, and training in income‐generating activities for economic empowerment
Worldwide Fistula Fund	Kenya	Psychosocial support, literacy and leadership training, and the development and facilitation of fistula solidarity groups—cooperative groups that identify an enterprise or activity for income generation

a Organizations provided included an adequate level of detail in the unpublished literature to abstract this information; other organizations may provide similar programming, but this information was not accessible during our review.

#### 3.4.2 Conference abstracts

Abstracts from five of seven ISOFS Conferences were accessible (Kenya 2009, Senegal 2010, Uganda 2014, Nigeria 2016, and Nepal 2018). Of 380 abstracts reviewed, 13 met inclusion criteria and were analyzed. Eligible abstracts described counseling, education, economic empowerment activities, and social support. Three abstracts reported on the Association for the Rehabilitation and Re‐orientation of Women for Development (TERREWODE) Uganda activities: Emasu^56^ described training in music, dance, and drama for community performance and education. Income earned enabled participation in savings and loan schemes. Ayotaru et al.^57^ presented TERREWODE's social reintegration model on social and economic well‐being as accounting for large variability in emotional well‐being of stakeholders. Tripathi et al.^58^ assessed TERREWODE participation among women with incurable fistula and found significant improvement in emotional distress, quality of life, and health satisfaction. Kabayambi et al.^59^ (Uganda) described a community participatory reflection approach including engaging community stakeholders to facilitate family counseling, financial assistance, and social group participation, which resulted in stigma mitigation and improved emotional status. In Pakistan, Syed^60^ presented on fistula management resulting in improved patient expectations and outlook. In Ethiopia, Fentaw et al.^61^ described an integrated adult learning program for women with inoperable fistula including basic literacy that resulted in further formal education, increased self‐confidence, and readiness for starting businesses. In Bangladesh, Mohiuddin et al.^62^ and Haque et al.^63^ described the process and performance of reintegration and rehabilitation through microcredit‐linked services including livelihood skills training, financial assistance for fistula clients and family members, and mobilization of local institutions. Outcomes were not provided; programmatic perspectives suggest the strategy is effective and efficient. In Burkina Faso, Ouedraogo et al.^64^ presented a medico‐surgical management intervention combined with social and economic support that resulted in increased social participation, resumption of sexual intercourse, and return to work. In Kenya, Pollaczek et al.^65^ and Mohamed and Pollaczek^66^ reported on Action on Fistula, a comprehensive initiative focused on expanding fistula care access including support groups to enhance social and psychological health and boost livelihood development opportunities. Enhanced follow‐up measures are implemented for longer‐term outcomes of continence status, social and psychological well‐being, and economic health. In Bangladesh, Rahman and Akhter^67^ presented results of financial support and training in income‐generating activities, basic literacy and numeric training, psychosocial counseling, accommodation in wait‐in centers, and medical support. Rehabilitated patients became community fistula advocates—starting new lives and gaining self‐confidence. Finally, Shallon et al.^68^ identified National Obstetric Fistula Strategic frameworks emphasizing multisectoral and multidisciplinary approaches, with exemplar countries: Nigeria, Uganda, Tanzania, and Guinea.

Review of programs from the three Global Maternal Newborn Health Conferences (New Delhi, 2010; Arusha, 2013; Mexico City, 2015) identified one eligible abstract. Traore and Maartens^69^ presented on the integration of family planning services into fistula care in Mali. Abstract books from two of the five Women Deliver conferences (Copenhagen, 2016; Vancouver, 2019) included no eligible abstracts.

## 4 DISCUSSION

To our knowledge this is the first study to review and consolidate published and unpublished literature on programming adjunct to female genital fistula surgery. Programming identified included various combinations of health education, physical therapy, social support, psychosocial counseling, and economic empowerment, and was based largely in sub‐Saharan Africa. Authors supported holistic approaches to reintegration programming, calling on resource expansion to meet the physical and psychosocial needs of women recovering from fistula. Additionally, strategies to improve feasibility, acceptability, and effectiveness of patient‐centered, informed intervention programming were identified.

Interventions addressed components of postsurgical social reintegration and rehabilitation needs demonstrated in the literature through education, counseling, physical rehabilitation, improving economic stability, and incorporating a full continuum of care approach.^17^, ^22^, ^27^, ^28^ However, no one program modeled the holistic approach recommended by authors. Implementation challenges included training, staffing, funding, and limited scope. Facility‐based program delivery was perceived as too limited in dose and breadth to have meaningful long‐term impacts; however, this strategy allows for efficient delivery. Data were limited to short‐term outcomes; only two primary studies assessed longer‐term outcomes, despite broad interest.^40^, ^45^

Implementation remains a major gap in the literature, including feasibility and outcomes. Only 11 studies were eligible and reported adequate detail on intervention components for inclusion within primary results; intervention dosage varied greatly. Despite recommendation of comprehensive programming, very few measured broad outcomes. Standardization of objective and patient‐reported outcomes would enable comparison across implementation approaches. In addition, while quality of study design was not evaluated formally, most studies were not controlled, and some reported no outcomes. While tested modalities align theoretically with improved outcomes, robust research design would allow testing of gains possible by program types and intensity, and inform cost‐effectiveness of various approaches.

The available evidence suggests positive outcomes result from several reintegration approaches. Physical therapy should be considered, both pre‐ and postoperatively, including strengthening exercises and instruction on postures and techniques to protect the pelvic floor.^27^, ^28^ Similarly, incorporation of evidence‐based psychosocial counseling modalities is critical. Future research should include controlled assessment of multicomponent interventions (e.g. health education, physical therapy, community and individual level psychosocial counseling, and economic empowerment), acknowledging the multilevel and multidomain nature of reintegration.^70^ In addition, efficient strategies for long‐term follow‐up and for integrating community and family linkage are needed.

Other challenges identified include a mismatch between the short‐term nature of the economic empowerment programming offered and women's needs; also seen in general poverty alleviation literature, some economic programming described was considered inadequate for substantial socioeconomic status improvement. Insufficient funding at multiple levels also hampers postrepair rehabilitation and reintegration, as it does prevention of fistula and other maternal morbidities. UNFPA cites a concerning decline overall in development assistance for maternal and newborn health.

Strengths of our study include our systematic protocol, our broad search strategies, and inclusion of unpublished literature. Despite finding a deficit of published information, our review synthesized the available outcomes and challenges prior studies have encountered and identified priorities for reintegration research. The lack of published articles and our language eligibility criteria may limit these findings. Additionally, no formal quality grading was conducted. As subsequent robust studies are implemented, a systematic review may become appropriate. Additionally, results were not reportable by fistula etiology, and the impact of changing etiology patterns on postrepair recovery and reintegration is unknown. Finally, the grey literature varied greatly in quality and detail; a formal landscape analysis including survey would provide a more comprehensive presentation of currently available programming.

The existing literature supports holistic fistula care including meeting postrepair physical, psychosocial, and economic needs of women through reintegration programming targeting short‐ and long‐term outcomes. However, the evidence base lacks robust research designs and systematic detailed reporting of intervention components and outcomes. We strongly encourage researchers and service providers to implement more robust evaluation designs and to broadly disseminate the results of their work so that the global fistula community can benefit through the development of best practices in reintegration.

## AUTHOR CONTRIBUTIONS

AE, JB, SO, and JKB conceived the project. AE, CP, and JB‐W drafted the protocol. AE, CP, AD, JB‐W, SO, JB, AK, and JK revised the protocol and agreed upon the final protocol version. AE, CP, RB, and LL reviewed the literature. AE, CP, and JB‐W drafted the manuscript. All authors revised the manuscript and approved the submitted version.

## CONFLICTS OF INTEREST

The authors have no conflicts of interest.

## Supporting information


**Table S1.** Search strategy for all databases. All searches were limited to January 1, 2000–July 8, 2019.
**Data S1.** List of organizational websites identified by the Campaign to End Fistula Partners (UNFPA)^1^ that were searched for relevant content.
