# Supplementary material for: Rehabilitation and reintegration programming adjunct to female genital fistula surgery: A systematic scoping review
Source: Int J Gynaecol Obstet. 2020 Jan 13;148(Suppl 1):42–58. doi: 10.1002/ijgo.13039 (PMC7003948; doi:10.1002/ijgo.13039)
Supplement: Supplementary file 1 — Table S1. Search strategy for all databases. All searches were limited to January 1, 2000–July 8, 2019. Data S1. List of organizational websites identified by the Campaign to End Fistula Partners (UNFPA)1 that were searched for relevant content. [file IJGO-148-42-s001.docx]

**Supporting information S1.** Search strategy for all databases. All searches were limited to January 1, 2000–July 8, 2019.

| **Name of database** | **Search strategy** |
| --- | --- |
| PubMed (1966–) | ("obstetric fistula"[tiab] OR "obstetric fistulae"[tiab] OR "vaginal fistula"[tiab] OR "vaginal fistulae"[tiab] OR "vesicovaginal fistula"[tiab] OR "vesicovaginal fistulae"[tiab] OR "vesico-vaginal fistulae"[tiab] OR "obstetric fistulas"[tiab] OR "vaginal fistulas"[tiab] OR "vesicovaginal fistulas"[tiab] OR "vesico-vaginal fistulas"[tiab] OR "Vaginal Fistula"[Mesh] OR "ureterovaginal fistula"[tiab] OR "rectovaginal fistula"[tiab] OR "genital tract fistula"[tiab] OR "urinary fistula"[tiab] OR OR "uro-vaginal fistula"[tiab] OR "recto-vaginal fistula"[tiab] OR "ureterovaginal fistulae"[tiab] OR "rectovaginal fistulae"[tiab] OR "genital tract fistulae"[tiab] OR "urinary fistulae"[tiab] OR "ureterovaginal fistulas"[tiab] OR "rectovaginal fistulas"[tiab] OR "genital tract fistulas"[tiab] OR "urinary fistulas"[tiab] OR "uro-vaginal fistulas"[tiab] OR "recto-vaginal fistulas"[tiab] OR "bladder fistula"[tiab] OR "bladder fistulae"[tiab] OR "bladder fistulas"[tiab] OR "cystocolic fistula"[tiab] OR "cystocolic fistulae"[tiab] OR "cystocolic fistulas"[tiab] OR "cystovaginal fistula"[tiab] OR "cystovaginal fistulae"[tiab] OR "cystovaginal fistulas"[tiab] OR "ureter fistula"[tiab] OR "ureter fistulae"[tiab] OR "ureter fistulas"[tiab] OR "urethra fistula"[tiab] OR "urethra fistulae"[tiab] OR "urethra fistulas"[tiab])  AND  (rehabilitation[tiab] OR "quality of life"[tiab] OR "Quality of Life"[Mesh] OR reintegration[tiab] OR rehabilitating[tiab] OR reintegrating[tiab] OR reintegrated[tiab] OR counseling[tiab] OR exercise[tiab] OR exercises[tiab] OR education[tiab] OR physiotherapy[tiab] OR knowledge[tiab] OR "self esteem"[tiab] OR "mental health"[tiab] OR "mental well-being"[tiab] OR psychological[tiab] OR behavioral[tiab] OR coping[tiab] OR depression[tiab] OR anxiety[tiab] OR suicidal[tiab] OR suicide[tiab] OR distress[tiab] OR stress[tiab] OR social[tiab] OR recovery[tiab] OR "skills training"[tiab] OR "gender-based"[tiab] OR power[tiab] OR "Power (Psychology)"[Mesh] OR empower[tiab] OR empowered[tiab] OR empowerment[tiab] OR livelihood[tiab] OR microfinance[tiab] OR financial[tiab] OR finance[tiab] OR "Socioeconomic Factors"[Mesh] OR "self help"[tiab] OR "self-help"[tiab] OR "Self Care"[Mesh] OR "Self-Management"[Mesh] OR training[tiab] OR mobility[tiab] OR independence[tiab] OR decision-making[tiab] OR respect[tiab]) |
| Embase (1947–) | (('obstetric fistula':ab,ti OR 'obstetric fistulae':ab,ti OR 'vaginal fistula':ab,ti OR 'vaginal fistulae':ab,ti OR 'vesicovaginal fistula':ab,ti OR 'vesicovaginal fistulae':ab,ti OR 'vesico-vaginal fistula':ab,ti OR 'vesico-vaginal fistulae':ab,ti OR 'obstetric fistulas':ab,ti OR 'vaginal fistulas':ab,ti OR 'vesicovaginal fistulas':ab,ti OR 'vesico-vaginal fistulas':ab,ti OR 'urinary tract fistula'/exp OR 'rectovaginal fistula'/exp OR 'ureterovaginal fistula':ab,ti OR 'rectovaginal fistula':ab,ti OR 'genital tract fistula':ab,ti OR 'urinary fistula':ab,ti OR 'uro-vaginal fistula':ab,ti OR 'recto-vaginal fistula':ab,ti OR 'ureterovaginal fistulae':ab,ti OR 'rectovaginal fistulae':ab,ti OR 'genital tract fistulae':ab,ti OR 'urinary fistulae':ab,ti OR 'uro-vaginal fistulae':ab,ti OR 'recto-vaginal fistulae':ab,ti OR 'ureterovaginal fistulas':ab,ti OR 'rectovaginal fistulas':ab,ti OR 'genital tract fistulas':ab,ti OR 'urinary fistulas':ab,ti OR 'uro-vaginal fistulas':ab,ti OR 'recto-vaginal fistulas':ab,ti OR 'bladder fistula':ab,ti OR 'bladder fistulae':ab,ti OR 'bladder fistulas':ab,ti OR 'cystocolic fistula':ab,ti OR 'cystocolic fistulae':ab,ti OR 'cystocolic fistulas':ab,ti OR 'cystovaginal fistula':ab,ti OR 'cystovaginal fistulae':ab,ti OR 'cystovaginal fistulas':ab,ti OR 'ureter fistula':ab,ti OR 'ureter fistulae':ab,ti OR 'ureter fistulas':ab,ti OR 'urethra fistula':ab,ti OR 'urethra fistulae':ab,ti OR 'urethra fistulas':ab,ti)  AND  (rehabilitation:ab,ti OR 'quality of life':ab,ti OR reintegration:ab,ti OR rehabilitating:ab,ti OR reintegrating:ab,ti OR reintegrated:ab,ti OR counseling:ab,ti OR exercise:ab,ti OR exercises:ab,ti OR education:ab,ti OR physiotherapy:ab,ti OR knowledge:ab,ti OR 'self esteem':ab,ti OR 'mental health':ab,ti OR 'mental well-being':ab,ti OR psychological:ab,ti OR behavioral:ab,ti OR coping:ab,ti OR depression:ab,ti OR anxiety:ab,ti OR suicidal:ab,ti OR suicide:ab,ti OR distress:ab,ti OR stress:ab,ti OR social:ab,ti OR recovery:ab,ti OR 'skills training':ab,ti OR 'gender-based':ab,ti OR power:ab,ti OR 'empowerment'/exp OR empower:ab,ti OR empowered:ab,ti OR empowerment:ab,ti OR livelihood:ab,ti OR microfinance:ab,ti OR financial:ab,ti OR finance:ab,ti OR 'self help':ab,ti OR 'self-help':ab,ti OR 'self care'/exp OR training:ab,ti OR mobility:ab,ti OR independence:ab,ti OR decision-making:ab,ti OR respect:ab,ti OR 'social status'/exp))  NOT  (cancer:ab,ti OR 'neoplasm'/exp OR prostate:ab,ti OR 'prostate'/exp OR hypospadia:ab,ti OR 'hypospadias'/exp OR penile:ab,ti OR transgender:ab,ti OR 'gender confirming':ab,ti) |
| Web of Science (1900–) | (("obstetric fistula" OR "obstetric fistulae" OR "vaginal fistula" OR "vaginal fistulae" OR "vesicovaginal fistula" OR "vesicovaginal fistulae" OR "vesico-vaginal fistula" OR "vesico-vaginal fistulae" OR "obstetric fistulas" OR "vaginal fistulas" OR "vesicovaginal fistulas" OR "vesico-vaginal fistulas" OR "ureterovaginal fistula" OR "rectovaginal fistula" OR "genital tract fistula" OR "urinary fistula" OR "uro-vaginal fistula" OR "recto-vaginal fistula" OR "ureterovaginal fistulae" OR "rectovaginal fistulae" OR "genital tract fistulae" OR "urinary fistulae" OR "uro-vaginal fistulae" OR "recto-vaginal fistulae" OR "ureterovaginal fistulas" OR "rectovaginal fistulas" OR "genital tract fistulas" OR "urinary fistulas" OR "uro-vaginal fistulas" OR "recto-vaginal fistulas" OR "bladder fistula" OR "bladder fistulae" OR "bladder fistulas" OR "cystocolic fistula" OR "cystocolic fistulae" OR "cystocolic fistulas" OR "cystovaginal fistula" OR "cystovaginal fistulae" OR "cystovaginal fistulas" OR "ureter fistula" OR "ureter fistulae" OR "ureter fistulas" OR "urethra fistula" OR "urethra fistulae" OR "urethra fistulas")  AND  (rehabilitation OR "quality of life" OR reintegration OR rehabilitating OR reintegrating OR reintegrated OR counseling OR exercise OR exercises OR education OR physiotherapy OR knowledge OR "self esteem" OR "mental health" OR "mental well-being" OR psychological OR behavioral OR coping OR depression OR anxiety OR suicidal OR suicide OR distress OR stress OR social OR recovery OR "skills training" OR "gender-based" OR power OR empower OR empowered OR empowerment OR livelihood OR microfinance OR financial OR finance OR "self help" OR "self-help" OR training OR mobility OR independence OR decision-making OR respect OR socioeonomic))  NOT  (cancer OR prostate OR hypospadia OR hypospadias OR penile OR transgender OR "gender confirming") |
| Popline (1970–) | ("obstetric fistula" OR "obstetric fistulae" OR "vaginal fistula" OR "vaginal fistulae" OR "vesicovaginal fistula" OR "vesicovaginal fistulae" OR "vesico-vaginal fistula" OR "vesico-vaginal fistulae" OR "obstetric fistulas" OR "vaginal fistulas" OR "vesicovaginal fistulas" OR "vesico-vaginal fistulas" OR "ureterovaginal fistula" OR "rectovaginal fistula" OR "genital tract fistula" OR "urinary fistula" OR "uro-vaginal fistula" OR "recto-vaginal fistula" OR "ureterovaginal fistulae" OR "rectovaginal fistulae" OR "genital tract fistulae" OR "urinary fistulae" OR "uro-vaginal fistulae" OR "recto-vaginal fistulae" OR "ureterovaginal fistulas" OR "rectovaginal fistulas" OR "genital tract fistulas" OR "urinary fistulas" OR "uro-vaginal fistulas" OR "recto-vaginal fistulas" OR "bladder fistula" OR "bladder fistulae" OR "bladder fistulas" OR "cystocolic fistula" OR "cystocolic fistulae" OR "cystocolic fistulas" OR "cystovaginal fistula" OR "cystovaginal fistulae" OR "cystovaginal fistulas" OR "ureter fistula" OR "ureter fistulae" OR "ureter fistulas" OR "urethra fistula" OR "urethra fistulae" OR "urethra fistulas")  AND  (rehabilitation OR "quality of life" OR reintegration OR rehabilitating OR reintegrating OR reintegrated OR counseling OR exercise OR exercises OR education OR physiotherapy OR knowledge OR "self esteem" OR "mental health" OR "mental well-being" OR psychological OR behavioral OR coping OR depression OR anxiety OR suicidal OR suicide OR distress OR stress OR social OR recovery OR "skills training" OR "gender-based" OR power OR empower OR empowered OR empowerment OR livelihood OR microfinance OR financial OR finance OR "self help" OR "self-help" OR training OR mobility OR independence OR decision-making OR respect OR socioeconomic) |
| PsycINFO (ProQuest, 1887–) | ("obstetric fistula" OR "obstetric fistulae" OR "vaginal fistula" OR "vaginal fistulae" OR "vesicovaginal fistula" OR "vesicovaginal fistulae" OR "vesico-vaginal fistula" OR "vesico-vaginal fistulae" OR "obstetric fistulas" OR "vaginal fistulas" OR "vesicovaginal fistulas" OR "vesico-vaginal fistulas" OR "ureterovaginal fistula" OR "rectovaginal fistula" OR "genital tract fistula" OR "urinary fistula" OR "uro-vaginal fistula" OR "recto-vaginal fistula" OR "ureterovaginal fistulae" OR "rectovaginal fistulae" OR "genital tract fistulae" OR "urinary fistulae" OR "uro-vaginal fistulae" OR "recto-vaginal fistulae" OR "ureterovaginal fistulas" OR "rectovaginal fistulas" OR "genital tract fistulas" OR "urinary fistulas" OR "uro-vaginal fistulas" OR "recto-vaginal fistulas" OR "bladder fistula" OR "bladder fistulae" OR "bladder fistulas" OR "cystocolic fistula" OR "cystocolic fistulae" OR "cystocolic fistulas" OR "cystovaginal fistula" OR "cystovaginal fistulae" OR "cystovaginal fistulas" OR "ureter fistula" OR "ureter fistulae" OR "ureter fistulas" OR "urethra fistula" OR "urethra fistulae" OR "urethra fistulas")  AND  (rehabilitation OR "quality of life" OR reintegration OR rehabilitating OR reintegrating OR reintegrated OR counseling OR exercise OR exercises OR education OR physiotherapy OR knowledge OR "self esteem" OR "mental health" OR "mental well-being" OR psychological OR behavioral OR coping OR depression OR anxiety OR suicidal OR suicide OR distress OR stress OR social OR recovery OR "skills training" OR "gender-based" OR power OR empower OR empowered OR empowerment OR livelihood OR microfinance OR financial OR finance OR "self help" OR "self-help" OR training OR mobility OR independence OR decision-making OR respect OR MAINSUBJECT.EXACT("Quality of Life") OR MAINSUBJECT.EXACT("Empowerment") OR MAINSUBJECT.EXACT("Interpersonal Control")) |
| Sociological Abstracts and Social Services Abstracts (ProQuest, 1963– and 1980–, searched together) | ("obstetric fistula" OR "obstetric fistulae" OR "vaginal fistula" OR "vaginal fistulae" OR "vesicovaginal fistula" OR "vesicovaginal fistulae" OR "vesico-vaginal fistula" OR "vesico-vaginal fistulae" OR "obstetric fistulas" OR "vaginal fistulas" OR "vesicovaginal fistulas" OR "vesico-vaginal fistulas" OR "ureterovaginal fistula" OR "rectovaginal fistula" OR "genital tract fistula" OR "urinary fistula" OR "uro-vaginal fistula" OR "recto-vaginal fistula" OR "ureterovaginal fistulae" OR "rectovaginal fistulae" OR "genital tract fistulae" OR "urinary fistulae" OR "uro-vaginal fistulae" OR "recto-vaginal fistulae" OR "ureterovaginal fistulas" OR "rectovaginal fistulas" OR "genital tract fistulas" OR "urinary fistulas" OR "uro-vaginal fistulas" OR "recto-vaginal fistulas" OR "bladder fistula" OR "bladder fistulae" OR "bladder fistulas" OR "cystocolic fistula" OR "cystocolic fistulae" OR "cystocolic fistulas" OR "cystovaginal fistula" OR "cystovaginal fistulae" OR "cystovaginal fistulas" OR "ureter fistula" OR "ureter fistulae" OR "ureter fistulas" OR "urethra fistula" OR "urethra fistulae" OR "urethra fistulas")  AND  (rehabilitation OR "quality of life" OR reintegration OR rehabilitating OR reintegrating OR reintegrated OR counseling OR exercise OR exercises OR education OR physiotherapy OR knowledge OR "self esteem" OR "mental health" OR "mental well-being" OR psychological OR behavioral OR coping OR depression OR anxiety OR suicidal OR suicide OR distress OR stress OR social OR recovery OR "skills training" OR "gender-based" OR power OR empower OR empowered OR empowerment OR livelihood OR microfinance OR financial OR finance OR "self help" OR "self-help" OR training OR mobility OR independence OR decision-making OR respect OR MAINSUBJECT.EXACT("Power") OR MAINSUBJECT.EXACT("Empowerment") OR MAINSUBJECT.EXACT("Social Power") OR MAINSUBJECT.EXACT("Quality of Life") OR MAINSUBJECT.EXACT("Rehabilitation")) |
| African Journals Online (2004–) | fistula AND (rehabilitation OR reintegration OR education OR empowerment OR empower OR "self help" OR "quality of life" OR recovery OR reintegrated OR reintegrating OR power OR training OR coping OR mental OR decision OR counseling OR distress OR social) |

**Supporting information S2.** List of organizational websites identified by the Campaign to End Fistula Partners (UNFPA)^1^ that were searched for relevant content.

- Aden Hospital (Yemen)
- African Medical and Research Foundation
- American College of Nurse-Midwives
- Babbar Ruga Fistula Hospital (Nigeria)
- Bangladesh Medical Association
- Bill & Melinda Gates Institute for Population and Reproductive Health
- Bugando Medical Center (United Republic of Tanzania) CARE
- Centers for Disease Control and Prevention (CDC)
- Centre Mère-Enfant (Chad)
- Centre National de Référence en Fistule Obstétricale (Niger)
- Centre National de Santé de la Reproduction & du Traitement des Fistules (Chad)
- Columbia University’s Averting Maternal Death and Disability Program (AMDD)
- Comprehensive Community Based Rehabilitation in Tanzania (CCBRT)
- CURE International Hospital of Kabul (Afghanistan)
- Direct Relief International
- Dr. Abbo’s National Fistula and Urogynaecology Center (Sudan)
- East Central and Southern Africa Association of Obstetrical and Gynecological Societies
- EngenderHealth
- Equilibres & Populations
- Eritrea Women’s Project
- Family Care International
- Fistula e.V.
- Fistula Foundation
- Fistula Foundation Nigeria
- Freedom from Fistula Foundation
- Friends of UNFPA
- Geneva Foundation for Medical Education and Research
- Girls’ Globe
- Governess Films
- Gynocare Fistula Center (Kenya)
- Hamlin Fistula (Ethiopia)
- Healing Hands of Joy (Ethiopia)
- Health and Development International
- Health Poverty Action (Sierra Leone)
- Hope Again Fistula Support Organization (Uganda)
- Human Rights Watch
- Institut de Formation et de Recherche en Urologie et Santé de la Famille (IFRU-SF) (Senegal)
- International Confederation of Midwives (ICM)
- International Continence Society
- International Federation of Gynecology and Obstetrics (FIGO)
- International Forum of Research Donors (IFORD)
- International Nepal Fellowship (INF)
- International Planned Parenthood Federation (IPPF)
- International Society of Obstetric Fistula Surgeons (ISOFS)
- International Urogynecological Association (IUGA)
- International Women’s Health Coalition, Islamic Development Bank
- Johns Hopkins Bloomberg School of Public Health
- Johnson & Johnson
- Kupona Foundation
- Lake Tanganyika Floating Health Clinic
- Ligue d’Initiative et de Recherche Active Pour la Santé et l’Education de la Femme (LIRASEF, Cameroon)
- London School of Hygiene and Tropical Medicine
- Maputo Central Hospital (Mozambique)
- Médecins du Monde
- Médecins Sans Frontières (MSF)
- Mercy Ships
- Moi University (Kenya)
- Monze Hospital (Zambia)
- Mulago Hospital/Medical School (Uganda)
- National Obstetric Fistula Centre, Abakiliki (Nigeria)
- Obstetrical and Gynecological Society of Bangladesh
- One by One
- Operation Fistula
- Pakistan National Forum on Women’s Health
- Pan African Urological Surgeons’ Association (PAUSA)
- Population Media Center
- Psychology Beyond Borders
- Regional Prevention of Maternal Mortality Network (RPMM, Ghana)
- Royal College of Obstetricians and Gynaecologists (RCOG)
- Sana’a Hospital (Yemen)
- Selian Fistula Project (United Republic of Tanzania)
- Société Africaine des Gynécologues-Obstétriciens (SAGO)
- Société Internationale d’Urologiel
- Solidarité Femmes Africaines (SOLFA)
- The Association for the Rehabilitation and Re-orientation of Women for Development (TERREWODE, Uganda)
- Uganda Childbirth Injury Fund
- United Nations Population Fund (UNFPA)
- United States Agency for International Development (USAID)
- University of Aberdeen
- University Teaching Hospital of Yaoundé (Cameroon)
- Virgin Unite
- White Ribbon Alliance
- Women and Health Alliance International (WAHA)
- Women’s Health Organization International (WHOI)
- Women’s Hope International (WHI)
- Women’s Missionary Society of the African Methodist Episcopal Church
- World Health Organization (WHO)
- World Vision
- Worldwide Fistula Fund
- Zonta International

^1^ UNFPA. The Maternal Health Thematic Fund. Phase II (2014-2017) - Progress Report. New York, NY: UNFPA; 2017.
